# Answers to Querists

**Published:** 1867-12

**Authors:** 


ANSWERS TO QUERISTS.

*Properties, Generation and Administration of Nitrous Oxide Gas.* Query 3d.—*Administration of Nitrous Oxide Gas? Answer.*

IF nitrous oxide gas is breathed for about thirty seconds and then taken away from the patient, violent muscular action attended with earnest declamation immediately follows the removal of the inhaling tube from the mouth.

When, however, this gas is breathed for one and a half to two minutes, complete repose attended with perfect anæsthesia ensues. It is easy to administer the gas until complete anæsthesis is produced; for after the first three or four inspirations the patient eagerly desires more, and all dread appears to have passed away.

"The dose of nitrous oxide necessarily depends upon the varied circumstances of race, age, sex, constitution, temperament, idiosyncrasy, and special application." "Thus for instance, as a general rule, persons of a lymphatic can bear larger quantities than those of a sanguine or bilious temperament; and these latter more than the nervous.—these being frequently very susceptible to its excitant influences." "Besides, as with most other agents, males will usually require more than females, adults than youths, and these again more than children." (*Zeigler.*)

In using this gas for dental operations, such as the extraction of teeth or the destruction of nerves of teeth, the patient should be seated in a suitable chair, which will admit of the back being lowered in cases of necessity.

A cork of the proper size, with a string attached to it, is placed between the jaws to prevent the closure of the mouth. The object of the string attached to the cork, is to guard against the unpleasant effects which might result, should the cork become displaced during the administration of the gas. A fatal case occurred in Philadelphia sometime since from carelessness in this respect; the cork slipped from between the teeth, and lodging in the throat caused death from suffocation.

Previous to the application of the inhaler or breathing tube, the patient is directed to take a full inspiration, followed by an exhalation, for the purpose of emptying the lungs as perfectly as possible of atmospheric air.

When this is done the mouth-piece of the inhaler is placed in the mouth and the patient requested to keep the lips tightly closed upon it. The nostrils are then held by an assistant, or where Clarke's rubber bag is used the fingers of the left hand press this bag closely over the nose and mouth about the mouth-piece of the inhaler, and at the same time support the breathing tube to which the inhaler is attached. The patient is then directed to take full inspirations. The first evidence of anæsthesia with the majority of patients, is snoring, like that of deep sleep.

To determine the proper time for operating, or as it has been termed the surgical period, the patient, previous to the administration of the gas, should be directed to raise the hand at every order of the operator.

Inability to make this motion is an evidence of the loss of voluntary power, which is soon succeeded by that of insensibility to pain.

As soon as the operation of extraction is performed, especially if the back teeth have been removed, the head of the patient should be inclined forward, or held over the spittoon, to prevent the blood from running down the throat, the cork removed from the mouth, which is readily done by means of the projecting portion of the string attached to it, and fresh air admitted into the room.

For the production of anæsthesia the inhalation of from four to eight gallons of the gas, will, in the majority of cases, prove sufficient. The gas can be inhaled from an India rubber bag, or from a tube leading directly from the gasometer, having attached to it a proper inhaler.

The method of inhaling the gas directly from the gasometer, is preferable in many respects to the use of the rubber bag, although some of the objections to the bag are obviated by having a proper inhaler attached to it.

It has been suggested when the operation is a protracted one, that the inhaler be removed from the mouth, fresh air admitted into the lungs, and the gas again administered without interrupting the anæsthetic condition.

The inhaler or breathing tube for the administration of nitrous oxide gas is constructed of vulcanized rubber or metal.

It consists of a tube and mouth-piece, the tube containing two valves, one valve upon the inside of the tube to allow the gas to pass through to the mouth of the patient; the other upon the outside to allow the exhalation to pass off and not be again inhaled. Attached to the tube is a stop-cock to arrest the flow of gas when desired.

A number of these inhalers are in use, known by the names of their inventors.

In certain conditions nitrous oxide gas, like other general anæsthetic agents, may produce dangerous and even fatal results, although it is considered safer than ether or chloroform. In disease of the heart, in active congestion, or acute inflammation of the brain, lungs or kidneys, or in a general plethoric condition, or where there is a tendency to a hemorrhagic diathesis, its use as an anæsthetic agent is attended with danger.

